# Superior digestive tract side effects after prolonged treatment with NSAIDs in patients with osteoarthritis

**Published:** 2015

**Authors:** OC Rogoveanu, CT Streba, CC Vere, L Petrescu, R Trăistaru

**Affiliations:** * Department of Medical Sciences II, Medical Rehabilitation, University of Medicine and Pharmacy of Craiova, Romania; ** Research Center for Gastroenterology and Hepatology, University of Medicine and Pharmacy of Craiova, Bucharest; *** Department of Medical Sciences I, Gastroenterology, University of Medicine and Pharmacy of Craiova, Bucharest; **** “Carol Davila” University of Medicine and Pharmacy, Bucharest, Romania

**Keywords:** NSAIDs, osteoarthritis, gastrointestinal side effects, ulcer, gastritis

## Abstract

**Introduction:** Anti-inflammatory drugs (NSAIDs) represent one of the classes of drugs commonly recommended for patients with osteoarthritis. These drugs have side effects that sometimes affect the digestive tract.

**Patients and Methods:** 30 patients with either axial or peripheral osteoarthritis, hospitalized between January 2013 and January 2015 in the Rehabilitation Clinic of the Clinical Emergency County Hospital of Craiova, were followed. All the patients included had upper gastrointestinal endoscopy performed, excluding patients who had a history of gastritis, endoscopically demonstrated ulcer or those with a history of gastrointestinal dyspeptic phenomena.

**Results:** The endoscopic examination found acute erosive gastritis in eight cases, gastric ulcer (four cases) and duodenal ulcer (six cases). No serious complications were encountered. Four patients had no clinical manifestations with endoscopic expression. Lesions were mainly mild esophagitis (Grade I) and only in three cases, esophagitis grade II or III was found. Twelve patients (40%) had Helicobacter pylori infection and had endoscopic changes (gastric ulcer, duodenal ulcer, or gastric lesions).

**Conclusions:** The adverse effects of NSAIDs in patients with osteoarthritis vary in severity. Patient age significantly influences the severity of injuries. We have not found significant differences in the incidence of COX2 selective and non-selective NSAID-induced lesions. Pathogenic interaction between the NSAID and Helicobacter Pylori infection is most often a synergistic effect in the development of gastritis or gastroduodenal ulcer.

## Introduction

Anti-inflammatory drugs (NSAIDs) represent one of the classes of drugs commonly recommended for patients with osteoarthritis [**1**]. In addition to the specific effect of improving the symptoms of painful and inflammatory conditions, these agents sometimes have significant side effects usually related to the dose and duration of administration [**2**–**6**].

The main adverse effects described in the upper digestive tract after prolonged administration of NSAIDs are:

- Esophagitis and esophageal strictures [**7**]

- Acute erosive gastritis and/ or bleeding [**4**,**5**]

- Gastric or duodenal ulcer, sometimes complicated by perforation [**5**]

- Upper gastrointestinal bleeding [**5**–**7**]

## Patients and Methods

Between January 2013 and January 2015, a group of 30 patients hospitalized in the Rehabilitation Clinic of the Emergency County Hospital of Craiova with either axial or peripheral osteoarthritis, were studied. Patient age ranged between 50 and 70 years with an equal distribution for both sexes. To relieve painful symptoms, patients received physical and NSAID therapy. Based on study protocols, only patients who required treatment with NSAIDs for more than 14 days (or by self-medication administration of NSAIDs at home, before admission) and having shown signs of digestive gastrointestinal phenomena, were selected.

Upper gastrointestinal endoscopy (UGE) was performed in all patients included in the study group in the Gastroenterology Clinic Clinical Emergency County Hospital Craiova and the laboratory or imaging investigations of the University of Medicine and Pharmacy of Craiova for objectifying changes induced by chronic treatment with NSAIDs.

Patients who had a history of gastritis or endoscopically demonstrated ulcer, as well as those with a history of gastrointestinal dyspeptic phenomena were excluded from the study.

COX2 selective NSAIDs were used in 10 of the patients while the other 20 were given non-selective NSAIDs. All patients were tested for Helicobacter pylori (HP) by serological analysis of the antibody and antigen detection in feces.

## Results and discussion

The pathogenesis of NSAID-induced gastrointestinal lesions requires the intervention of several local and systemic mechanisms [**8**–**10**].

The local effect of these drugs has the following main components:

- *The interaction between NSAIDs and surface phospholipids* in relation to the detergent properties. This is the mechanism through which erosions and acute gastrointestinal bleeding, often hemodynamically significant, most frequently occur.

NSAIDs with acid structure cannot biochemically dissociate in the intragastric acid environment and remain in lipophilic form, which facilitates their penetration through the mucus layer on the surface of the gastric epithelium. The interaction with the phospholipid membrane surface of the epithelial cells leads to loss of the hydrophobic capacity and favors retrodiffusion of the H + ions (the main cause of ulcers) [**10**].

- *Local vascular ischemia* - occurs early after the administration of NSAIDs and is due to the adverse effect of NSAIDs and ionized H + which causes lesions of the small vessels of the corium submucosa. Capillary thromboses occur, affecting the local defense mechanisms of the gastroduodenal mucosal [**10**,**11**].

- *“Ion capturing” and depletion of ATP* - within the intracellular environment of the stomach, NSAIDs dissociates in H + and the ionic form that cannot leave the cell and remains sequestered. Both intracellular components have toxic effects either by the absence of the coupling of oxidative phosphorylation, or by inhibiting the respiratory chain [**10**]. The consequence is the decline in local, intracellular, stocks of ATP, altered intercellular junctions, H + ions retro diffusion fostering and facilitating effect of endogenous aggressive factors (HCl, pepsin, lysolecithin) involved in ulcers [**10**,**12**].

- *Systemic effects* of inhibition of cyclooxygenases 1 and 2 are the main mechanism of action of NSAIDs [**12**–**14**]. The decrease in prostaglandin synthesis causes an impairment of local defense mechanisms of the gastric mucosa with a decreased local production of mucus and reduced local microcirculation blood flow.

Cyclooxygenase 1 is involved in the production of prostaglandins in the gastric protective inhibit gastric acid secretion, stimulates the synthesis of mucous and bicarbonate and the immune cell function in the stomach lining [**10**,**15**].

Cyclooxygenase 2 has local and systemic proinflammatory effects. Therefore, it follows that the use of selective NSAIDs (COX-2 inhibitors) has, at least theoretically, less risk of developing mucosal lesions in the upper digestive tract [**15**,**16**].

Endoscopic studies on the enrolled patients showed esophageal lesions, gastric and/ or duodenal ulcers.

**Fig. 1:**
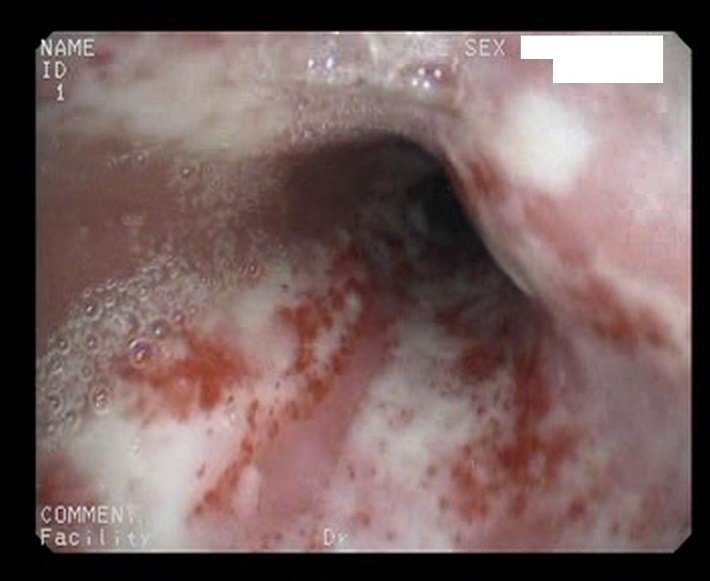
Endoscopic view of esophagitis in an elderly patient self-medicating with NSAIDs for gonarthrosis

Eight patients had esophagitis (**Fig. 1**) and esophageal strictures changes. 

The eight patients were aged between 60 and 68 years and constantly used non-selective NSAIDs for a long time, mainly as self-medication for coxarthrosis or gonarthrosis. Lesions were mainly mild esophagitis (Grade I) and, esophagitis was found in only three cases grade II or III. These patients had a lower pressure on the lower esophageal sphincter at manometry, which correlated with the inhibition of local prostaglandin synthesis resulted in esophageal mucosal lesions.

Local inflammation caused by acid reflux is followed by healing with excessive synthesis of collagen fibers, which leads to esophageal strictures [**17**]. Gastroduodenal lesions are the most common side effects of NSAIDs long used by patients with arthritis.

**Fig. 2:**
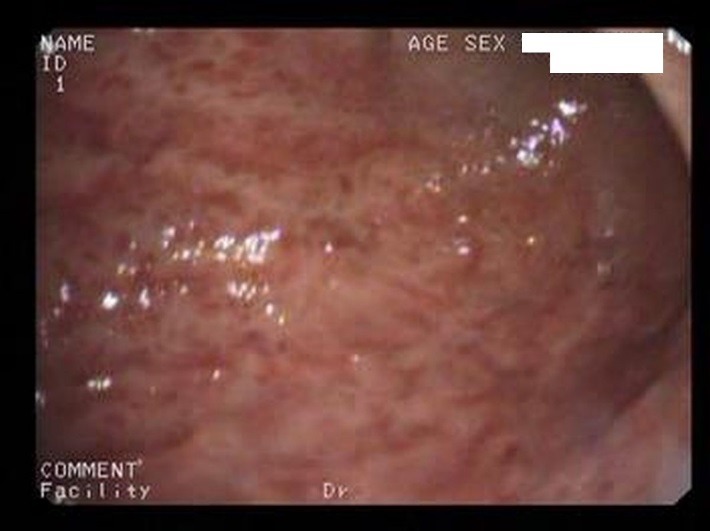
Acute erosive gastritis changes in a patient who underwent prolonged COX2 NSAIDs therapy for osteoarthritis

**Fig. 3:**
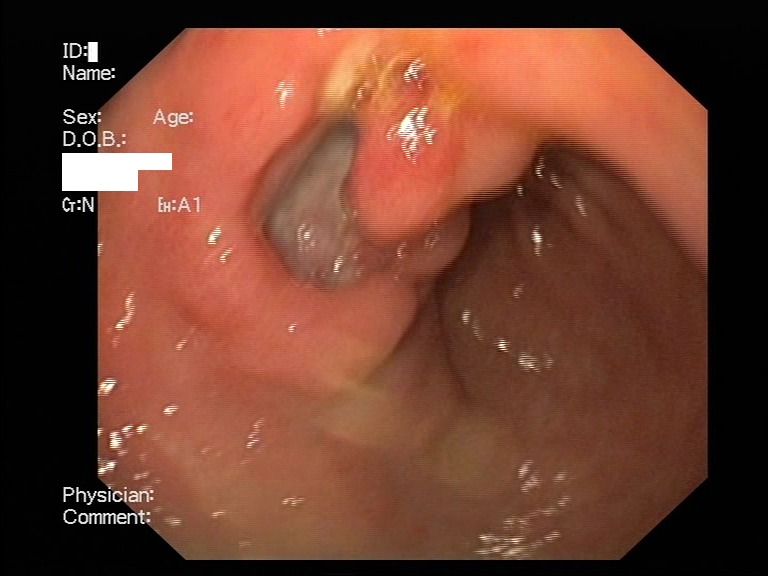
Gastric ulcer in a Helicobacter Pylori positive patient and chronic NSAID user who presented with suggestive clinical symptoms

**Fig. 4:**
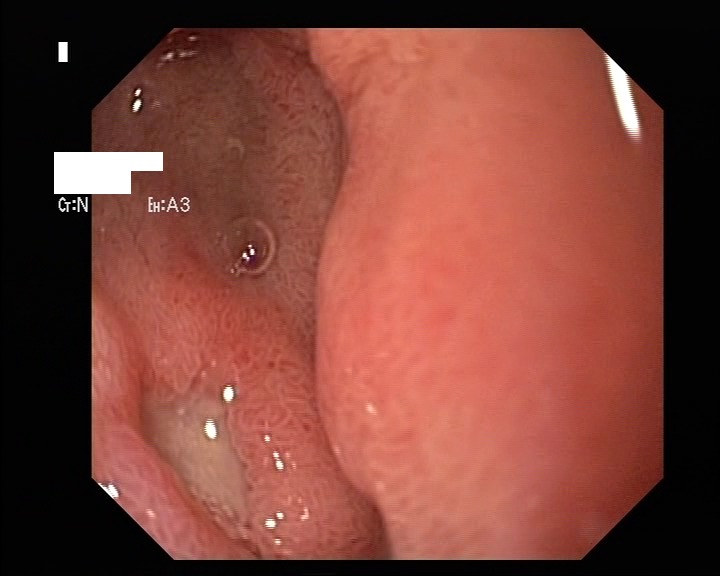
Duodenal ulcer (endoscopic aspect) encountered in an elder patient after NSAID therapy

Acute erosive gastritis changes present in eight cases, gastric ulcer (four cases) and duodenal ulcer (six cases) were found within the study group – **Fig. 2**-**4**. No serious, life-threatening, complications were encountered such as upper gastrointestinal bleeding or a type of hollow organ perforation. Four of the patients who displayed clinical manifestations (heartburn, belching, acid regurgitation) had no endoscopic expression, so they were classified as functional dyspepsia.

Most studies published so far assessed the advanced age of patients as being the main risk factor for NSAID induced gastrointestinal complications [**14**–**18**].

There are several hypotheses on the role of comorbidities or other medication associated with specific diseases of this age, but it seems however that the main mechanism is the decreased regeneration ability of mucosal defense in the elderly [**18**].

There are substantial differences between gastroduodenal tolerability of various classes of NSAIDs used in practice. An inverse correlation between COX2 selective NSAIDs and digestive complications was suggested. In our study, we did not find these differences, endoscopic changes being encountered in the 10 patients who were treated with COX2 selective NSAIDs [**15**,**16**].

However, we have found a direct correlation between the doses, timing of drug use and damage to the digestive tract. The four patients with functional dyspepsia sporadically administered the NSAIDs, only during hyperalgesia episodes of arthrosis and always under the protection of proton pump inhibitors, even though the administering period exceeded 3 weeks. The other 26 patients in whom endoscopic examination revealed disease changes were constant users of NSAID close to the maximum dose, even if they concomitantly administered gastric protection medication.

Pathogen interactions between HP infection and consumption of NSAIDs in the digestive tract lesions are currently arousing much controversy [**9**]. In our study, 12 patients (40%) were infected with HP and had endoscopic changes at the gastric or duodenal level (gastric ulcer, duodenal ulcer, or gastric lesions)

There are a number of common mechanisms by which NSAIDs and HP contribute to digestive tract lesions, even though they have divergent mechanisms [**7**–**9**]. Both factors inhibit the secretion of mucus and bicarbonate (gastric mucosal protective factors). However, the effect on local microvasculature is different. NSAIDs decrease local blood flow by reducing prostaglandin synthesis while HP infection does not affect this mechanism. However, experimental studies have shown that the gastric and duodenal mucosa regeneration is much better after administering therapy for eradicating the HP infection [**9**].

## Conclusions

NSAID adverse effects seen in patients with osteoarthritis who use this medication for long periods vary in severity from functional digestive phenomena to gastric and duodenal ulcer.

The pathogenic mechanisms are local and systemic but all target local factors aimed at the defense of the digestive tract (mucus and decreasing the synthesis of prostaglandins, altered intercellular junctions favoring H + ions retrodiffusion, microthrombosis in local circulation, etc.).

Patient age significantly influences the severity of NSAID-induced lesions due to lower defense capability and regeneration of the digestive tract, comorbidities, and other associated treatments.

In our study, we found no significant differences in the incidence of NSAID-induced lesions of both COX2 selective and nonselective variants, although references in literature showed a less aggressive potential of selective COX2 inhibitors on the gastrointestinal mucosa.

Pathogen interactions between NSAIDs and HP infection are mostly synergistic in the development of gastritis or peptic ulcer, a fact sustained by the faster recovery of the integrity of the digestive mucosa after eradication therapy.
